# Cognitive mediators of US—China differences in early symbolic arithmetic

**DOI:** 10.1371/journal.pone.0255283

**Published:** 2021-08-25

**Authors:** John E. Opfer, Dan Kim, Lisa K. Fazio, Xinlin Zhou, Robert S. Siegler

**Affiliations:** 1 Department of Psychology, The Ohio State University, Columbus, Ohio, United States of America; 2 Psychology and Human Development, Vanderbilt University, Nashville, Tennessee, United States of America; 3 The Siegler Center for Innovative Learning, The State Key Laboratory of Cognitive Neuroscience and Learning, Beijing Normal University, Beijing, China; 4 Department of Human Development, Columbia University-Teachers College, New York, New York, United States of America; French National Center for Scientific Research (CNRS) & University of Lyon, FRANCE

## Abstract

Chinese children routinely outperform American peers in standardized tests of mathematics knowledge. To examine mediators of this effect, 95 Chinese and US 5-year-olds completed a test of overall symbolic arithmetic, an IQ subtest, and three tests each of symbolic and non-symbolic numerical magnitude knowledge (magnitude comparison, approximate addition, and number-line estimation). Overall Chinese children performed better in symbolic arithmetic than US children, and all measures of IQ and number knowledge predicted overall symbolic arithmetic. Chinese children were more accurate than US peers in symbolic numerical magnitude comparison, symbolic approximate addition, and both symbolic and non-symbolic number-line estimation; Chinese and U.S. children did not differ in IQ and non-symbolic magnitude comparison and approximate addition. A substantial amount of the nationality difference in overall symbolic arithmetic was mediated by performance on the symbolic and number-line tests.

## Introduction

Mathematics proficiency differs greatly among nations [1–3], with a large advantage for East Asian over US students [4]. This “learning gap” appears before formal schooling, and it grows with each year [5].

Many differences in educational inputs exist that might explain the advantage of East Asian children. These differences include a transparent base-10 naming system for numbers [6], better quality math instruction in East Asian schools [7, 8], more time spent on math instruction [4], greater math knowledge of East Asian teachers [9], and more positive attitudes about math among East Asian students and parents [4, 10].

What remains unclear, however, is *how* these differences in environmental and educational inputs (e.g., language, instruction methods, time on task, teacher quality, and attitudes) lead to differences in outputs (e.g., addition skill). One possibility is that these national differences contribute *directly* to differences in math proficiency; this possibility appears to be the tacit assumption of most educational research. Another possibility is that national differences contribute mostly *indirectly*, by means of producing math-related cognitive gains, such as in general intelligence or basic numeracy.

Our ignorance of the second possibility may put us in a situation like that of 19th century physicians who treated scurvy with citrus fruits but had not yet discovered the role of ascorbic acid. We know *that* the input matters but not *how*. This is a potentially dangerous situation. Knowing only that citrus fruits were important, physicians came to treat scurvy by boiling lime juice (which destroys ascorbic acid), were unable to explain why some fruits were ineffective at treating scurvy, overlooked non-obvious sources of ascorbic acid (such as fresh meat), and ultimately abandoned the treatment of scurvy with citrus fruits [11]. What doctors needed to identify was the *mediator* by which citrus fruits exercised their effects.

Without identifying mediators between educational inputs and student math proficiency, we are likely to make mistakes similar to those of 19^th^ century physicians. Similar to treating scurvy with boiled lime juice, less successful schools might imitate the practices of more successful ones without achieving similar results, due to a lack of understanding of the processes that produced the superior results. In contrast, identifying factors that mediate increases in math proficiency offers a promising path forward. Such knowledge could indicate how successful math outcomes can be achieved even without (for example) Chinese number naming, Chinese cultural attitudes toward mathematics, Chinese teachers’ deep understanding of the math being taught, and so on.

In this study, we explore how cognitive abilities, especially numerical magnitude knowledge, mediate nation effects on math performance, in particular symbolic arithmetic. A model for this approach comes from research on the effects of SES (socioeconomic status) on differences in math proficiency. It has long been known that children in higher-income families are more proficient in mathematics than children in lower-income ones [12], but the mechanism has been unclear. In what turned out to be crucial, children from higher-income families have been shown to have better numerical magnitude knowledge [13]. The relation proved to be causal as well as correlational. When programs were implemented to promote numerical magnitude knowledge in low-income children, the advantage for children from high-income children shrank [14] or disappeared [15]. Thus, by discovering important cognitive differences between high and low SES families—and targeting them for intervention—researchers were able to close an important learning gap. No previous study, however, has examined cognitive mechanisms through which cross-national differences lead to differences in math achievement.

### Numerical magnitude knowledge as a potential mediator of nation effects

As with the gap between children in high and low SES families, the gap between East Asian and US children might emerge through activities affecting quality of numerical magnitude knowledge [16]. In this view, practices that improve the abilities to compare numerical magnitudes, to add them approximately, and to order them on a linearly-increasing “mental number line” might be key factors in producing early symbolic arithmetic.

In previous studies, many measures of numerical magnitude knowledge have been shown to correlate with math achievement. Accuracy in identifying the more numerous of two sets of objects predicts math achievement test scores from preschool to adulthood [17–19]. Ability to find the numerically greater of two Arabic numerals or number words even more strongly predicts arithmetic proficiency [20, 21]. Accurately estimating the location on a number-line of Arabic numerals or the numbers of object in arrays also positively predicts math skills [22], with the predictive power again being greater for symbolic than non-symbolic number-line estimation [21]. All of these relations hold after statistically controlling for relevant variables, including IQ, reading comprehension, and SES [20, 22].

The same tests of numerical magnitude knowledge often show an advantage for East Asian children relative to Western peers. Chinese children’s accuracy in numerical magnitude comparison [23] and number line estimation [24] exceeds that of Western age peers. Chinese children also show the same logarithmic-to-linear shift in number-line estimation as their Western peers, but they typically generate linear estimation patterns at younger ages [24, 25].

### Current study

**Fig 1:**
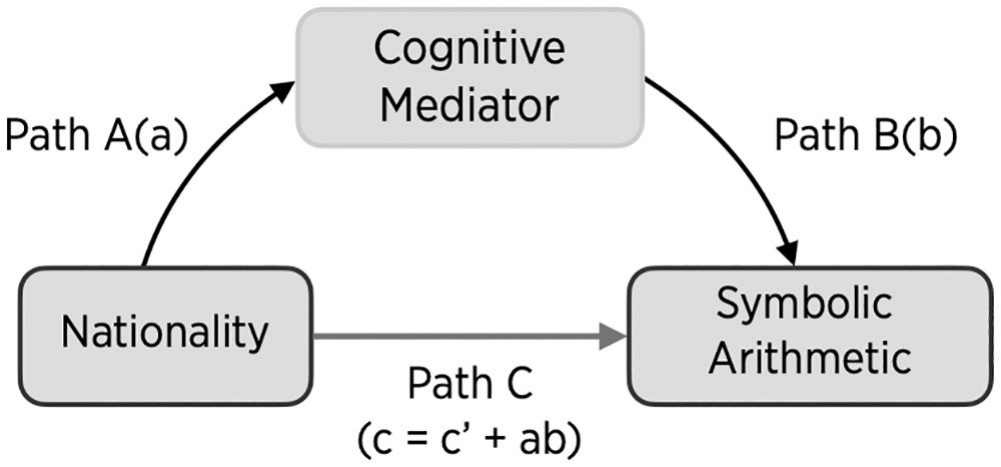
Mediation model. Mediation model of the nationality effect on symbolic arithmetic. The total effect of nationality (c) is comprised of the direct effect (c’) and the indirect effect through a mediator (ab).

The literature reviewed above suggests a simple model in which the quality of numerical magnitude knowledge mediates the effect of nationality on symbolic arithmetic (Fig 1). The model predicts: (1) American children will attain lower arithmetic scores than their East Asian peers, (2) numerical magnitude knowledge will be associated with better symbolic arithmetic, (3) American children will demonstrate less numerical magnitude knowledge than their East Asian peers, and (4) the effect of nation on symbolic arithmetic scores and errors will be mediated by numerical magnitude knowledge. The current study was designed to test all four predictions. We also examined whether national differences in magnitude knowledge are limited to symbolic numbers (which must be learned through cultural experiences) or extend to non-symbolic numerosity (which can be perceived fairly accurately by human infants and non-human animals).

## Method

### Participants

Participants were 95 kindergartners recruited at the same time of year from four schools in Beijing, China and in Columbus, OH serving predominantly middle SES populations. No differences were present in means or variability of ages of participants in the United States (*n* = 41; *M* = 5.46 years, *SD* = 0.22; 46% female and 54% male; 78% White, 10% Black, and 7% Asian, 5% biracial) and China (*n* = 54; *M* = 5.52 years, *SD* = 0.23; 54% female and 46% male; 100% Asian). A power analysis revealed that this sample size was sufficient to detect an effect size of 0.50 (Cohen’s *d*, [26]) with power 0.80 for national differences in a t-test. Power analyses using the Sobel test also indicated that the sample size has adequate power for simple and multiple mediations analyses (power>.92 for simple mediation and power>.91 for multiple mediation analyses) [27–29]. Informed written consent was obtained from all participants’ parents or legal guardians. Participants were given a set of tasks, whose materials and procedure were approved by the Institutional Review Board of The Ohio State University and of Beijing Normal University.

### Tasks

#### Symbolic arithmetic assessment

The symbolic arithmetic test was a speeded paper-and-pencil addition test based on Geary et al. [5]. The test comprised 50 items, ranging from 1+1 to 5+5, with each problem given twice. Children were allowed 1 min to solve as many problems as possible. The reliability of this test, assessed in terms of Cronbach’s alpha, was very high (.95).

#### Coding

The coding task was based on that used by Stevenson et al. [30] and is similar to that found in intelligence tests given to children and adults. The code comprised nine paired symbols. One symbol was always a simple figure, such as ⋀ or ⊔, and the other symbol was always a numeral (i.e., 1–9). After the child was guided through seven practice items of the code, the child was given 2 minutes to complete as many of the 133 test items as possible. One of the non-numeric symbols was presented on each trial; the child was to write the associated numeral below it.

#### Non-symbolic number comparison

**Fig 2:**
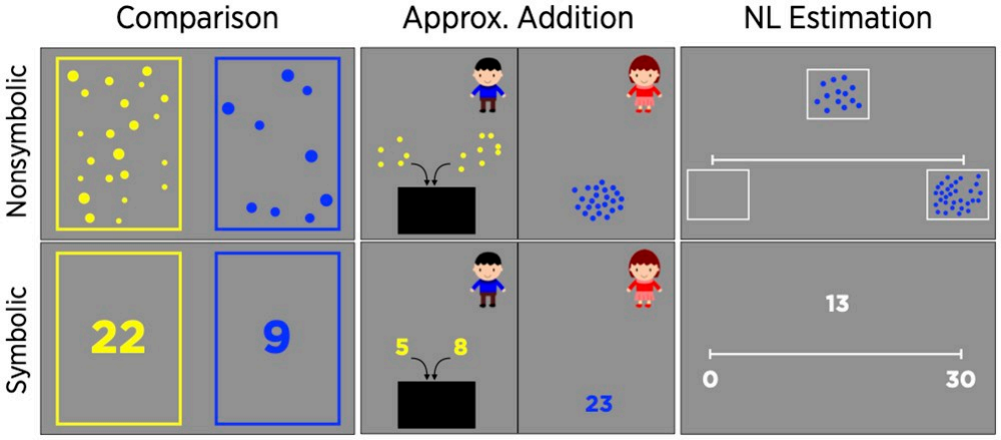
Numerical magnitude tasks. Illustration of non-symbolic and symbolic number tasks.

The non-symbolic magnitude comparison task was administered using free software downloaded from http://www.panamath.org; it was similar to the comparison task in Halberda et al. [31] (Fig 2). On each trial, yellow and blue dots were presented on the left and right side of screen, respectively, for 2000ms, followed by a backward mask displayed for 200ms. Children were then asked to choose which side had more dots as quickly and accurately as possible. There were four ratio bins (1.22–1.41, 1.41–1.63, 1.70–1.96, and 2.63–3.04), 14 trials in each bin, resulting in 56 trials. Ratios were defined as the larger number divided by the smaller number. For example, for a comparison between 17 yellow and 21 blue dots, the ratio was 1.24, whereas for a comparison between 8 yellow and 22 blue dots, the ratio was 2.75. The array on each side of the screen included 4 to 30 dots. Dot size was controlled on half of the trials, and total area was controlled on the other half. Therefore, children needed to base their responses on the number of dots, not on the size or the total area of dots, to respond consistently correctly. There were 2 practice trials prior to the task. The reliability of the task, based on Cronbach’s alpha, was.88.

#### Non-symbolic approximate addition

The non-symbolic approximate addition task was based on Barth et al. [32] (Fig 2). Children were required to add two dot arrays and compare the sum of dots to a third dot array. On each trial, two arrays of yellow dots appeared and then moved behind the left side of a screen. This was followed by an array of blue dots that appeared on the right side of a screen. Children were asked to judge whether yellow or blue dots were more numerous. Each array had 4 to 30 dots. To prevent counting, total display time was brief, 2500ms. Stimuli were generated from three ratio bins (1.22–1.41, 1.41–1.63, and 1.70–1.93). There were 8 trials per ratio bin (24 trials in total). On half of the trials, the sum of the addends was larger than the comparison. The total area was negatively correlated with the number of dots on half of the trials; it was positively correlated with number on the other half. Cronbach’s alpha was.84.

#### Non-symbolic number-line estimation

This task paralleled the number-line estimation task with Arabic numerals used in Kim and Opfer [33] (Fig 2). Children were presented a number-line with a box of 30 dots on the right end and an empty box of dots on the left end. The endpoints were labeled as “a lot of dots” and “no dots”. The 20 arrays that children estimated had 5, 6, 7, 8, 9, 10, 11, 12, 13, 14, 17, 18, 19, 20, 21, 22, 23, 27, 28, or 29 dots), which matched the numbers in the symbolic number-line task. Each dot array was presented above the number-line for 2000ms. On half of the trials, the size (area) of individual dots was held constant, with the cumulative area of dots increasing with number; on the other half of the trials, the cumulative area of dots was held constant, with the size of individual dots decreasing with number. These controls were designed to assess whether children based their responses on area instead of number. Children were told to focus on the number of dots rather than their sizes, and were asked to mouse-click the location of the number of dots on the number-line. Cronbach’s alpha was.73.

#### Symbolic number comparison

In this task, children judged the relative magnitudes of two Arabic numerals. The numbers ranged from 4 to 30 and matched those in the non-symbolic magnitude comparison task (Fig 2). Two numerals were presented on the screen for 2000ms; they then were covered with a mask for 200ms. There were 58 trials: 2 practice trials and then 14 trials randomly sampled from each of 4 ratio bins: 1.22–1.41, 1.41–1.63, 1.70–1.96, and 2.63–3.04. As in the non-symbolic number comparison task, the instructions emphasized the importance of both speed and accuracy. Cronbach’s alpha was.90.

#### Symbolic approximate addition

The symbolic approximate addition task was identical to the non-symbolic approximate addition task except that stimuli were Arabic numerals (Fig 2). On each trial, children saw a computer-animated event, narrated by a researcher, in which a number appeared and moved behind a screen. After viewing two numerals (e.g., 9 and 12) move sequentially behind the screen, children saw a third numeral (e.g., 25) and judged whether the sum of the first two numbers or the third number was greater. Presentation time was equal to that on the non-symbolic task (2500ms), making it unlikely that the kindergartners could enumerate and add these numbers quickly enough to obtain exact sums. (As we will see, Chinese kindergartners required a mean of 12s to solve easier arithmetic problems exactly.) Across trials, the sum and comparison arrays differed by varying ratios (1.22–1.41, 1.41–1.63, and 1.70–1.93); each was larger on half the trials. On the 24 test problems, the sum of the addends ranged from 4 to 30, as did the comparison numbers. Cronbach’s alpha was.85.

#### Symbolic number-line estimation

This task was identical to the non-symbolic number-line estimation task except that Arabic numerals were substituted for dot arrays (Fig 2). For example, each number-line was flanked by the numbers “0” and “30” at each end. Children were asked to mouse-click a place where a given number belonged. The reliability of the task based on Cronbach’s alpha was.86.

### Procedure

The experimental tasks were administered in a quiet space in schools over three sessions. Tasks and items within tasks were given in random order. Each session took less than 15 min, and children were tested individually.

## Results

The results are organized using the logic of a mediational analysis (Fig 1). In the first section, "Nationality effect on early symbolic arithmetic," we estimate the total effect of nationality on children’s arithmetic proficiency (Path C). This effect (*c*) is the sum of the direct effect (*c’*) of nationality on arithmetic scores (controlling for mediating variables) and the indirect effect (*ab*) of nationality through mediating variables. Analyses in the next section, "Potential mediators of nationality effects on early symbolic arithmetic" separate direct from indirect effects. These analyses test the effect of nationality on each potential mediator (Path A), of each potential mediator on arithmetic proficiency (Path B), and how much of the full effect of nationality on symbolic arithmetic is explained by mediation via each potential mediator. Finally, in the last section, "Cognitive mechanisms underlying nationality effects on early symbolic arithmetic," we conduct a multiple mediation analysis to examine how much of the nationality effect on arithmetic proficiency occurs through the effect of numerical magnitude and which other cognitive mechanisms result in cross-national differences in symbolic arithmetic.

### Nationality effect on early symbolic arithmetic

**Fig 3:**
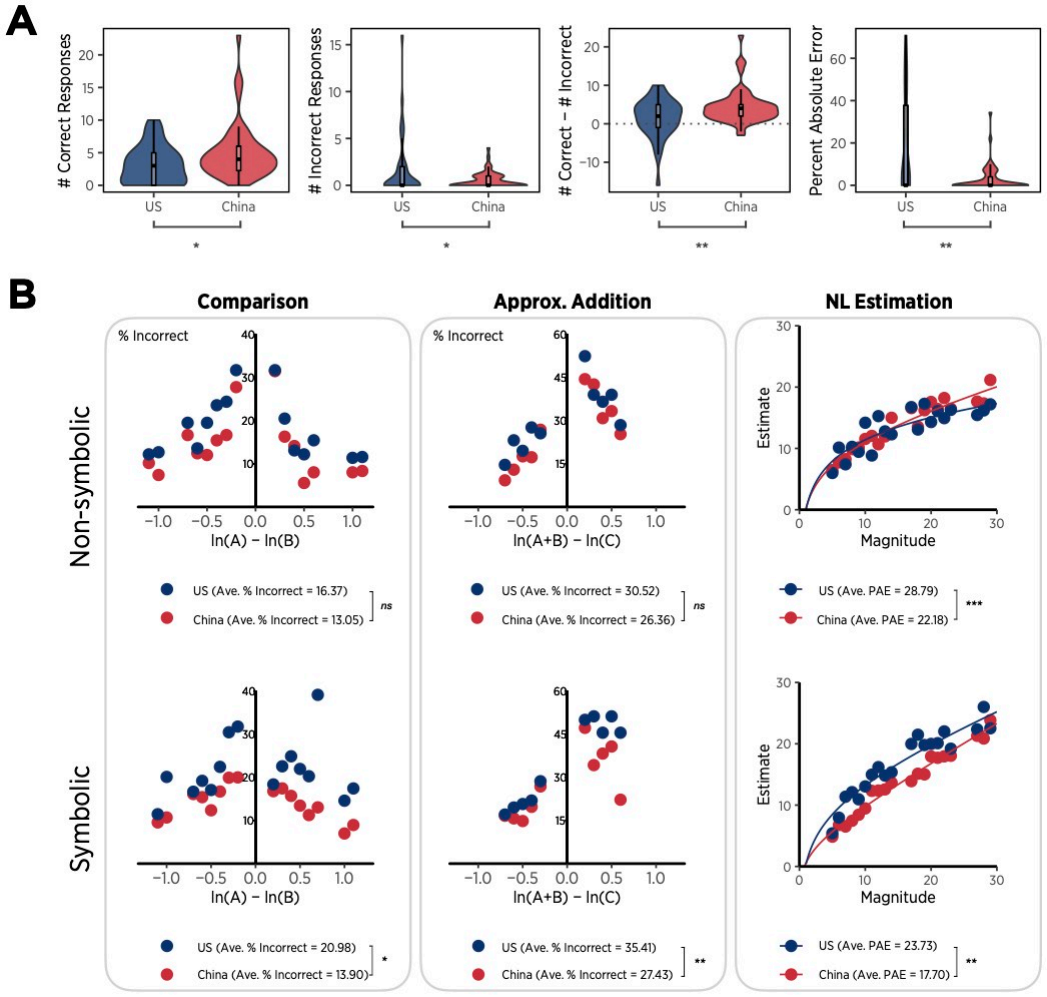
Performance on symbolic arithmetic and numerical magnitude measures. A. Violin plots and boxplots of the four measures of symbolic arithmetic. For each boxplot, the central mark represents the median, the hinges are the first and third quartiles (i.e., the 25^th^ and 75^th^ percentiles), and the whiskers extend from the hinges to the smallest or largest values that are within 1.5×interquartile range (IQR) from hinges—i.e., 1^st^ quartile-1.5×IQR to 3^rd^ quartile+1.5×IQR. B. Performance on numerical magnitude measures: the mean error rates of comparison and approximate addition, and median responses in number-line estimation.

As shown in Fig 3A, accuracy on the standardized arithmetic test differed by nationality: Chinese kindergartners (*M* = 5.02, *SD* = 4.20) provided more correct answers than US kindergartners (*M* = 3.17, *SD* = 2.87), *t*(93) = 2.42, *p* < .05, *d* = .50, and US children (*M* = 1.61, *SD* = 3.20) provided more incorrect answers than Chinese children (*M* = .50, *SD* = .84), *t*(93) = -2.44, *p* < .05, *d* = .51. The arithmetic scores, the difference between the number of correct and incorrect responses, were roughly three times as high in Chinese kindergartners (*M* = 4.52, *SD* = 4.50) as in US kindergartners (*M* = 1.56, *SD* = 5.00), *t*(93) = 3.03, *p* < .01, *d* = .63. Finally, on trials where children erred, the difference between the incorrect response that was advanced and the correct answer measured by the percent absolute error (PAE = 100×|response-correct answer|/correct answer) was smaller among Chinese children (*M* = 2.95, *SD* = 6.05) than US peers, (*M* = 16.42, *SD* = 23.86), *t*(88) = -3.97, *p* < .001, *d* = .85. Thus, compared to Chinese kindergartners, US kindergartners appeared less likely to provide correct arithmetic facts, more likely to answer incorrectly, and more likely to make large errors.

To ensure that the national differences in symbolic arithmetic were not driven by outliers, we excluded data that were 3 standard deviations (SD) below or above the mean for each arithmetic measure. This led to exclusion of 3 Chinese children for the number of correct answers, 2 US children for the number of incorrect answers, 1 Chinese and 1 US children for arithmetic score, and 3 US children for arithmetic PAEs. For the PAE measure, 5 additional US children who did not provide a single response and thus had zero errors were removed. After the outlier exclusion, it turned out that Chinese children (*M* = 4.24, *SD* = 2.61) provided more correct answers than US children (*M* = 3.17, *SD* = 2.87), but the difference was marginally significant, *t*(90) = 1.86, *p* = .07, *d* = .39. Also, US children (*M* = 1.05, *SD* = 1.89) provided marginally more incorrect answers than Chinese children (*M* = .50, *SD* = .84), *t*(91) = -1.90, *p* = .06, *d* = .40. The arithmetic scores were significantly higher in Chinese kindergartners (*M* = 4.17, *SD* = 3.73) than in US kindergartners (*M* = 2.00, *SD* = 4.19), *t*(91) = 2.63, *p* < .01, *d* = .55. Finally, PAEs in symbolic arithmetic were smaller among Chinese children (*M* = 2.95, *SD* = 6.05) than US children, (*M* = 11.97, *SD* = 19.41), *t*(85) = -3.18, *p* < .01, *d* = .70. Although all the arithmetic measures reveal a tendency that Chinese kindergartners outperformed US kindergartners, arithmetic scores and PAEs provide stronger evidence that symbolic arithmetic differs between the two nations. To examine the relations between nationality and symbolic arithmetic more in depth, we use the two measures without outliers for further analyses below.

### Potential mediators of nationality effects on early symbolic arithmetic

#### Coding

The first potential mediator that we examined was a numeric task with no requirement for magnitude judgments. This Coding task (WISC-III; also known as Digit-Symbol Substitution task, WAIS-III) yields scores (0–133) that are widely used to assess performance IQ of children [34, 35]. The task is thought to index information processing speed [30] and to be more strongly correlated with arithmetic performance than other IQ subscales [36].

Performance on this task did not differ between Chinese (*M* = 15.56, *SD* = 6.43) and US kindergartners (*M* = 14.34, *SD* = 8.13), *t*(93) = .81, *ns*, suggesting that our samples were equivalent to those in previous studies and national norms. Variability also did not differ between scores of children in the two nations, *F*(40, 53) = 1.60, *ns*. Although IQ scores correlated with overall arithmetic proficiency (*r*(93) = .60, *p* < .001), it seemed highly unlikely that the nationality effect on symbolic arithmetic could be mediated by IQ (because IQ differences were minimal). However, we conducted and report a mediation analysis to illustrate the statistical process.

To know whether (and how strongly) IQ mediates nationality effects on symbolic arithmetic, we used Preacher and Hayes’ [37] PROCESS procedure and bootstrapped mediation analysis to test the significance of mediation effects. When arithmetic scores were used, the total effect of nationality on symbolic arithmetic was 2.17. Controlling for IQ reduced the direct effect of nationality to 1.93, which remained significantly different from zero (95% CI [.50, 3.36]). In contrast, the remaining indirect effect of nationality via IQ was.24, which did not differ from zero (95% CI[-.56, 1.16]). The indirect effect through IQ accounts for only 11% of the total effect of nationality on symbolic arithmetic (.24/2.17). When PAEs in the arithmetic test were submitted to the identical mediation analysis, the results remained similar. The most nationality effects on arithmetic PAEs took place directly through nationality itself (-9.34, 95% CI[-14.87, -3.82]) with the small indirect effect of nationality via IQ (.31, 95% CI[-.85, 2.29]). The indirect effect accounts for only -3% of the total effect of nationality (.31/-9.03).

#### Non-symbolic magnitude tasks

**Fig 4:**
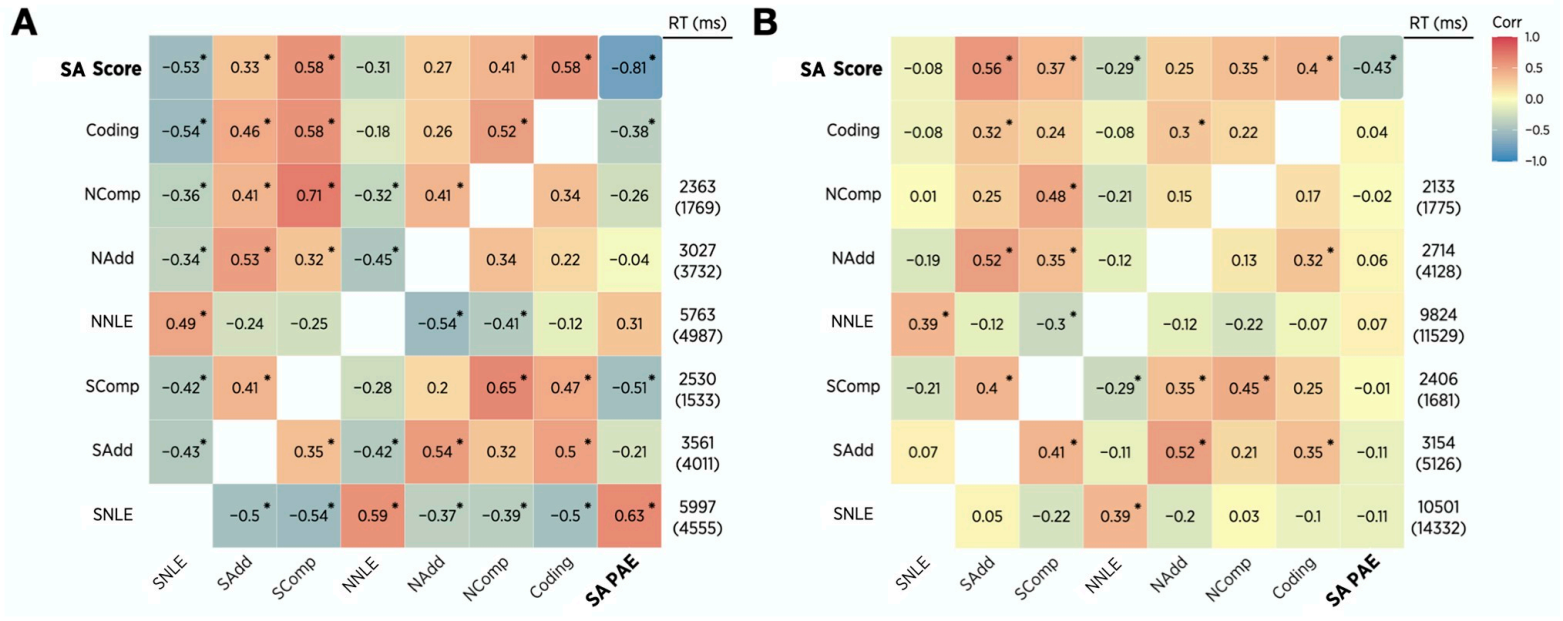
Means and standard deviations (in parenthesis) of reaction times and correlations among symbolic arithmetic (SA) scores and PAEs, accuracy of coding, and accuracy of numerical magnitude tasks in US (A) and Chinese (B) children. NComp represents non-symbolic comparison, NAdd non-symbolic approximate addition, NNLE non-symbolic number-line estimation, SComp symbolic comparison, SAdd symbolic approximate addition, and SNLE symbolic number-line estimation. For number-line estimation tasks, percent absolute errors were used as an accuracy measure. Correlation coefficients on the upper diagonal were computed using data after removing outliers based on arithmetic scores (n = 93), whereas those on the lower diagonal were from data with outlier exclusion by symbolic arithmetic PAEs (n = 87). * indicates *p* < .05.

*Non-symbolic number comparison*. We next examined the effect of nationality on non-symbolic number comparison, using three measures that have been claimed to track math proficiency and/or the psychophysics of numerosity perception. These measures were accuracy [38, 39], Weber fraction [31, 40], and the numeric distance effect [41]. For outlier exclusion, the mean and standard deviation of reaction times of each child were computed for each numerical magnitude task (Fig 4). Responses that were slower than 2.5 SDs above the mean RT were excluded from analyses in number comparison and all other tasks (3.5% of all data). Across all three indices of non-symbolic number comparison, we observed no differences between Chinese and US children. Overall accuracy scores (0–100%) for Chinese (*M* = 86.95, *SD* = 11.81) and US children (*M* = 83.63, *SD* = 15.00), did not differ, *t*(93) = 1.21, *ns*. The average Weber fraction for Chinese (*M* = .31, *SD* = .58) and US children (*M* = .43, *SD* = .72) also did not differ, *t*(93) = -.94, *ns*. Finally, both US and Chinese children’s accuracy reliably increased as the distance of the logarithms of compared numbers increased (Fig 3B). The distance effect (i.e., slope of accuracy against ratio) for Chinese children (*b* = -15.62, *R*^*2*^ = .49) again did not differ from that of US children (*b* = -16.61, *R*^*2*^ = .66), *F*(1, 12) = .01, *ns*. Accuracy of non-symbolic comparison did not show an association with arithmetic PAEs (*r*(85) = -.15, *p*>.05), but with arithmetic scores (*r*(91) = .39, *p* < .001); however, the indirect effect of nationality on arithmetic scores by means of non-symbolic number comparison was.33, which accounts for only 15% of the total effect (.33/2.17). This indirect effect did not differ from zero (95% CI[-.29, 1.10]). Similarly, the indirect effect of nationality on arithmetic PAEs through non-symbolic comparison was.01, which is not different from zero (95% CI[-1.36,.99]) and accounts for less than -1% of the total nationality effect (.01/-9.03).

*Non-symbolic approximate addition*. The quality of non-symbolic approximate addition (a + b > or <c) was examined using the same indices as non-symbolic number comparison. Again, across all indices, no nation effects were observed. Accuracy scores (0–100%) did not differ for Chinese (*M* = 73.64, *SD* = 13.4) and US children (*M* = 69.48, *SD* = 17.02), *t*(93) = 1.33, *ns*. Average Weber fractions were also similar for Chinese (*M* = .74, *SD* = .76) and US children (*M* = 1.04, *SD* = .99), *t*(93) = -1.64, *ns*. Finally, both US and Chinese children’s accuracy increased with the distance of the logarithms of the sum and the comparison (Fig 3B). The distance effect was not different between Chinese (*b* = -63.23, *R*^*2*^ = .94) and US children (*b* = -60.39, *R*^*2*^ = .84), *F*(1, 8) = .03, *ns*. Although accuracy of non-symbolic approximate addition showed a positive correlation with arithmetic scores (*r*(91) = .28, *p* < .01), the indirect effect of nationality on arithmetic scores by means of non-symbolic number addition again was as low as.24 (.24/2.17 = 11%) and statistically non-significant (95% CI[-.12,.98]). Accuracy of non-symbolic approximate addition was not correlated with arithmetic PAEs (*r*(85) = -.04, *p*>.05), and the indirect effect of nationality on arithmetic PAEs via non-symbolic addition was not different from zero (-.02, 95% CI[-1.08,.72]), explaining less than 1% of nationality effects (-.02/-9.03).

*Non-symbolic number-line estimation*. Like the two numerical magnitude tasks, estimates of numerical magnitudes on a number line could be assessed with Percent Absolute Error as well as by obtaining parameter values for psychometric functions linking the numeric stimuli to the spatial judgment [42–44]. We evaluated a number of competing psychometric functions for this purpose, including log-linear [45, 46], one-cycle power, and two-cycle power models [47]. Consistent with previous reports testing the logarithmic and linear models separately against these models in the context of symbolic number-line estimation [44, 48], the combined log-linear model provided a better fit to median estimates than the one-cycle power (ΔAICc = 29.76; probability of log-linear = 99.99%) and two-cycle power models (ΔAICc = 54.96; probability of log-linear = 99.99%). For this reason, we used the λ value of the log-linear model, which tracks the degree of logarithmicity in children’s estimates, to provide a psychophysical characterization of numeric estimation.

Errors in Chinese children’s estimates (PAE = 100×|estimate-given magnitude|/upper bound magnitude, *M* = 22.18, *SD* = 6.73) were smaller than errors in US children’s estimates (PAE, *M* = 28.79, *SD* = 7.90), *t*(93) = -4.40, *p* < .0001, *d* = .91. Moreover, the median estimates by Chinese children were less logarithmic (λ = .63) than those by US children (λ = .95) (Fig 3B). Finally, when λ scores were computed for individual children’s estimates, the mean λ score for US children (*M* = .85, *SD* = .30) was higher than for Chinese children (*M* = .70, *SD* = .37), *t*(93) = -2.07, *p*<05, *d* = .43. Errors in non-symbolic number-line estimation were negatively correlated with arithmetic scores (*r*(91) = -.37, *p* < .001), indicating that more accurate estimates accompanied better symbolic arithmetic performance. More importantly, we found that the indirect effect differed from zero, 1.04 (95% CI[.42, 2.08]), and that 48% (1.04/2.17) of the nationality effect on arithmetic scores arose indirectly through non-symbolic number-line estimation. When arithmetic PAEs were used for symbolic arithmetic, there was a positive association between arithmetic PAEs and errors in non-symbolic number-line estimation (*r*(85) = .30, *p* < .01). Also, the indirect effect of nationality through non-symbolic number-line estimation was different from zero, -2.14 (95% CI[-5.74, -.17]), which equals 24% of the total nationality effect (-2.14/-9.03).

#### Symbolic magnitude tasks

*Symbolic number comparison*. Symbolic number comparison also differed between Chinese and US children. Percent correct was greater for Chinese children (*M* = 86.10, *SD* = 13.67) than US children (*M* = 79.02, *SD* = 16.56), *t*(93) = 2.28, *p* < .05, *d* = .47. The average Weber fraction was smaller among Chinese children (*M* = .34, *SD* = .61) than US children (*M* = .64, *SD* = .89), although values for children in the two nations differed only marginally, *t*(93) = -1.96, *p* = .05, *d* = .41. Both US and Chinese children became more accurate with increasing distance between the logarithms of the numbers being compared; the magnitude of the distance effect did not differ between Chinese (*b* = -10.93, *R*^*2*^ = .89) and US children (*b* = -10.71, *R*^*2*^ = .53), *F*(1, 12) = .002, *ns*. Accuracy of symbolic number comparison predicted arithmetic scores (*r*(91) = .50, *p* < .001). Further, we found that the indirect effect was substantial,.75 (95% CI[.01, 1.76]), indicating that 35% (.75/2.17) of the nationality effect on symbolic arithmetic was an indirect effect mediated through symbolic number comparison. Accuracy of symbolic number comparison was also associated with arithmetic PAEs (*r*(85) = -.32, *p* < .01), but the indirect effect on arithmetic PAEs did not differ from zero, -.92 (95% CI[-4.31,.36]), accounting for 10% of nationality effects.

*Symbolic approximate addition*. Across most indices of symbolic approximate addition, Chinese children outperformed US children. Overall accuracy (0–100%) was greater for Chinese (*M* = 72.57, *SD* = 13.97) than for US children (*M* = 64.59, *SD* = 14.46), *t*(93) = 2.72, *p* = .008, *d* = .56. The mean Weber fraction was lower for Chinese (*M* = .88, *SD* = .83) than for US children (*M* = 1.30, *SD* = .96), *t*(93) = -2.30, *p* < .05, *d* = .48. Finally, both US and Chinese children’s accuracy reliably increased with the distance of the logarithms of the numbers being compared, though the distance effect did not differ between Chinese (*b* = -53.92, *R*^*2*^ = .87) and US children (*b* = -52.76, *R*^*2*^ = .84), *F*(1, 8) = .006, *ns*. Accuracy of symbolic approximate addition correlated positively with arithmetic scores (*r*(91) = .49, *p* < .001). Further, the indirect effect was significant,.92 (95% CI[.21, 1.99]), which meant that 42% (.92/2.17) of the nationality effect on arithmetic scores arose indirectly through symbolic approximate addition. Similarly, accuracy of symbolic approximate addition presented a negative correlation with arithmetic PAEs (*r*(85) = -.22, *p* < .05). The indirect effect on arithmetic PAEs was different from zero, -.99 (95% CI[-2.84, -.11]), which indicates that about 11% of the nationality effect (-.99/-9.03) occurred indirectly through symbolic approximate addition.

*Symbolic number-line estimation*. As with non-symbolic number-line estimation, the combined log-linear model provided a better fit to median estimates than the one-cycle power model (ΔAICc = 23.78; probability of log-linear = 99.99%) and two-cycle power model (ΔAICc = 44.72; probability of log-linear = 99.99%). Additionally, the proportion of children’s estimates best fit by the log-linear model was also higher than those fit by the one-cycle power model (log-linear, 64%; 1CPM, 36%), and two-cycle power model (log-linear, 87%; 2CPM, 13%). For this reason, we again used the λ value of the log-linear model.

As with other measures of symbolic numeric knowledge, Chinese children’s accuracy of symbolic number-line estimates (PAE, *M* = 17.70, *SD* = 8.32) was greater than US children’s accuracy (PAE, *M* = 23.73, *SD* = 10.44), *t*(93) = 3.13, *p* < .01, *d* = .65. Chinese children’s median estimates were less logarithmic (λ = .25) than those in US children (λ = .55). Finally, when λ scores were computed for individual children’s estimates, the mean λ score for US children (*M* = .75, *SD* = .32) was higher than for Chinese children (*M* = .47, *SD* = .37), *t*(93) = -3.86, *p* < .001, *d* = .80. Thus, consistent with previous reports [25], US kindergartners’ symbolic number line estimates were less accurate and more logarithmic than those of Chinese children. Because amount of error in symbolic estimates was negatively associated with arithmetic scores (*r*(91) = -.36, *p* < .001), and because estimation errors were greater among US children, symbolic number-line estimation accuracy was expected to mediate the relation between nationality and symbolic arithmetic. Consistent with this hypothesis, the indirect effect via symbolic number-line estimation differed from zero,.71 (95% CI[.12, 1.76]); 33% (.71/2.17) of the nationality effect on arithmetic scores was accounted for indirectly through symbolic number-line estimation. Errors in symbolic number-line estimation were also positively correlated with arithmetic PAEs (*r*(85) = .44, *p* < .001). The indirect effect through symbolic estimation was different from zero, -2.78 (95% CI[-8.00, -.28]), explaining 31% of national effects on arithmetic PAEs (-2.78/-9.03).

### Cognitive mechanisms underlying nationality effects on early symbolic arithmetic

Previously, independent mediation analyses demonstrated that nation effects on symbolic arithmetic were partially mediated by a variety of cognitive abilities, including non-symbolic number-line estimation, symbolic number comparison, symbolic approximate addition, and symbolic number-line estimation. Because these cognitive abilities were correlated with each other, the total indirect effect of nationality on symbolic arithmetic that occurred through these cognitive abilities was not equivalent to the sum of the partial effects. To control for collinearity, multiple mediation analyses were conducted to estimate how much of the effect of nationality on symbolic arithmetic was explained by indirect effects through the cognitive abilities, and which mechanisms uniquely mediated relations between nation and symbolic arithmetic. For easier interpretation of the results, we used error rates (100—percent correct) instead of percent correct for comparison and approximate addition tasks. This linear transformation did not change the mediation results but allowed the measures to denote inaccuracy similar to the PAE in number-line tasks.

**Fig 5:**
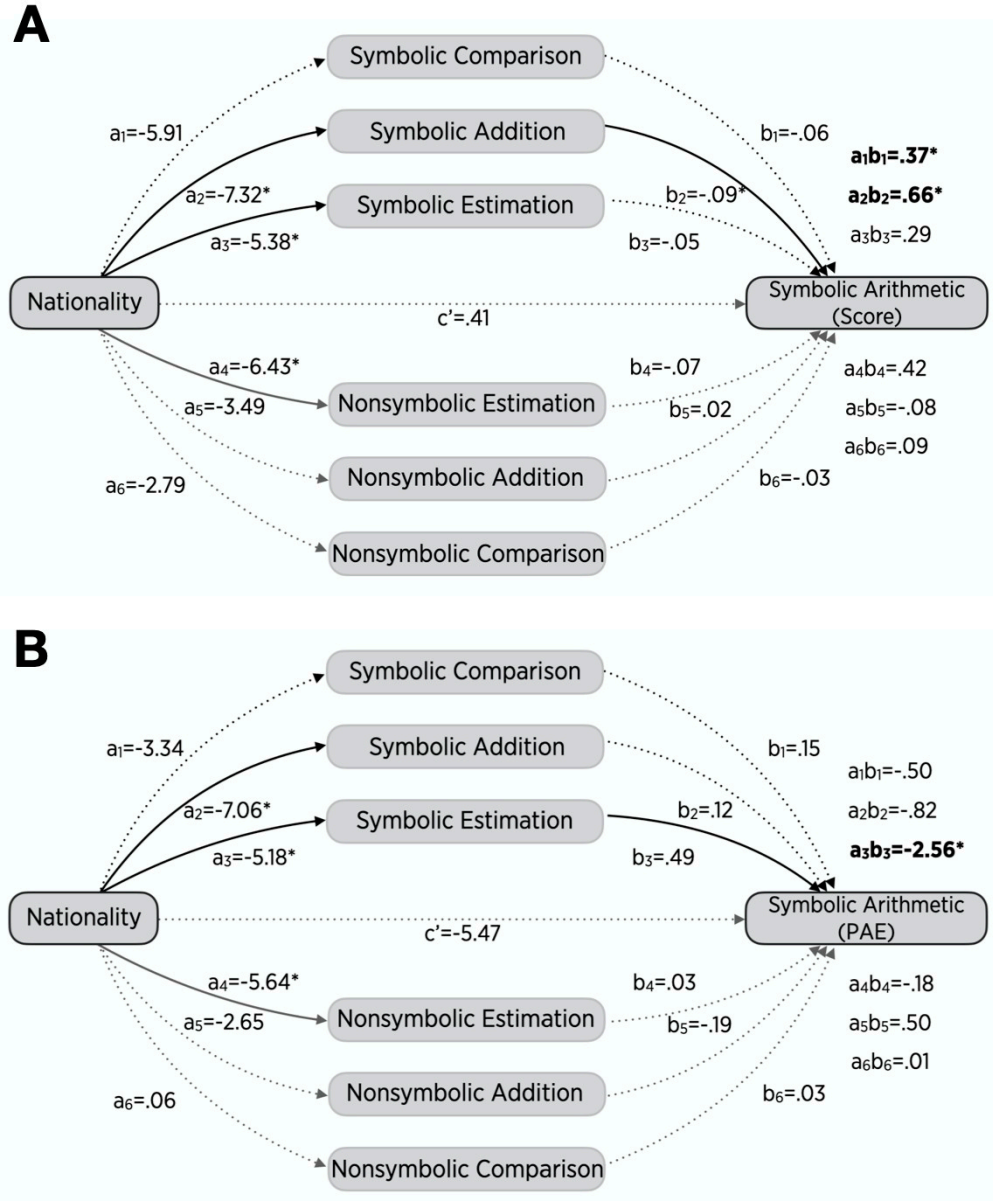
Multiple mediation analyses with arithmetic scores (A) and arithmetic PAEs (B) as an outcome variable. Solid and dotted lines represent significant and non-significant relations between variables, respectively. Results imply that a substantial amount of the nationality effects may occur through the numerical magnitude tasks (a_1_b_1_+a_2_b_2_+a_3_b_3_+a_4_b_4_+a_5_b_5_+a_6_b_6_). Only a non-significant proportion of the total effects (c’) was explained by nationality itself.

We found that only 19% of the total effects of nationality on arithmetic scores were directly from nationality itself (c’ = .41, 95% CI[-1.13, 1.95]); 81% of the total effects were mediated by numeric magnitude abilities (∑ab = 1.76, 95% CI[.66, 3.28], Fig 5A). When arithmetic PAEs were used as an outcome variable for symbolic arithmetic, 61% of nationality effects on symbolic arithmetic occurred directly from nationality itself, but the direct effect was not different from zero (c’ = -5.47, 95% CI[-11.23,.28]); 39% of the total effects took place indirectly through numeric magnitude abilities, and the mediation effect was different from zero (∑ab = -3.55, 95% CI[-9.15, -.11], Fig 5B). Thus, if the total effect of nation on symbolic arithmetic is the magnitude of the "learning gap" between Chinese and US children, a substantial amount of this learning gap may take place by means of numerical magnitude knowledge.

To explore underlying mechanisms in more detail, we tested the contributions of the combination of symbolic numerical magnitude tasks that were significant in the multiple medication analysis and of the combination of symbolic and spatial tasks that were significant in simple mediation analyses. The mediation of the three symbolic numeric measures accounted for 61% of nationality effects on symbolic arithmetic scores and 43% of nationality effects on arithmetic PAEs. Compared to non-symbolic numeric measures, however, their mediation effects were not significantly greater (∑ab = 1.32, 95% CI[-.26, 2.46] for arithmetic scores; ∑ab = -3.88, 95% CI[-11.02,.29] for arithmetic PAE). The combination of symbolic-numeric and spatial-numeric judgments (i.e., three symbolic numeric judgments and non-symbolic number-line estimation) accounted for 81% of the total effects on arithmetic scores and 45% of the total effects on arithmetic PAEs. The indirect effects through symbolic-numeric and spatial-numeric tasks were significantly greater than those of the combination of non-symbolic and non-spatial tasks (∑ab = 1.74, 95% CI[.49, 3.41] for arithmetic scores; ∑ab = -4.06, 95% CI[-10.91, -.02] for arithmetic PAEs).

### Symbolic arithmetic as a mediator between nationality and cognitive abilities

**Fig 6:**
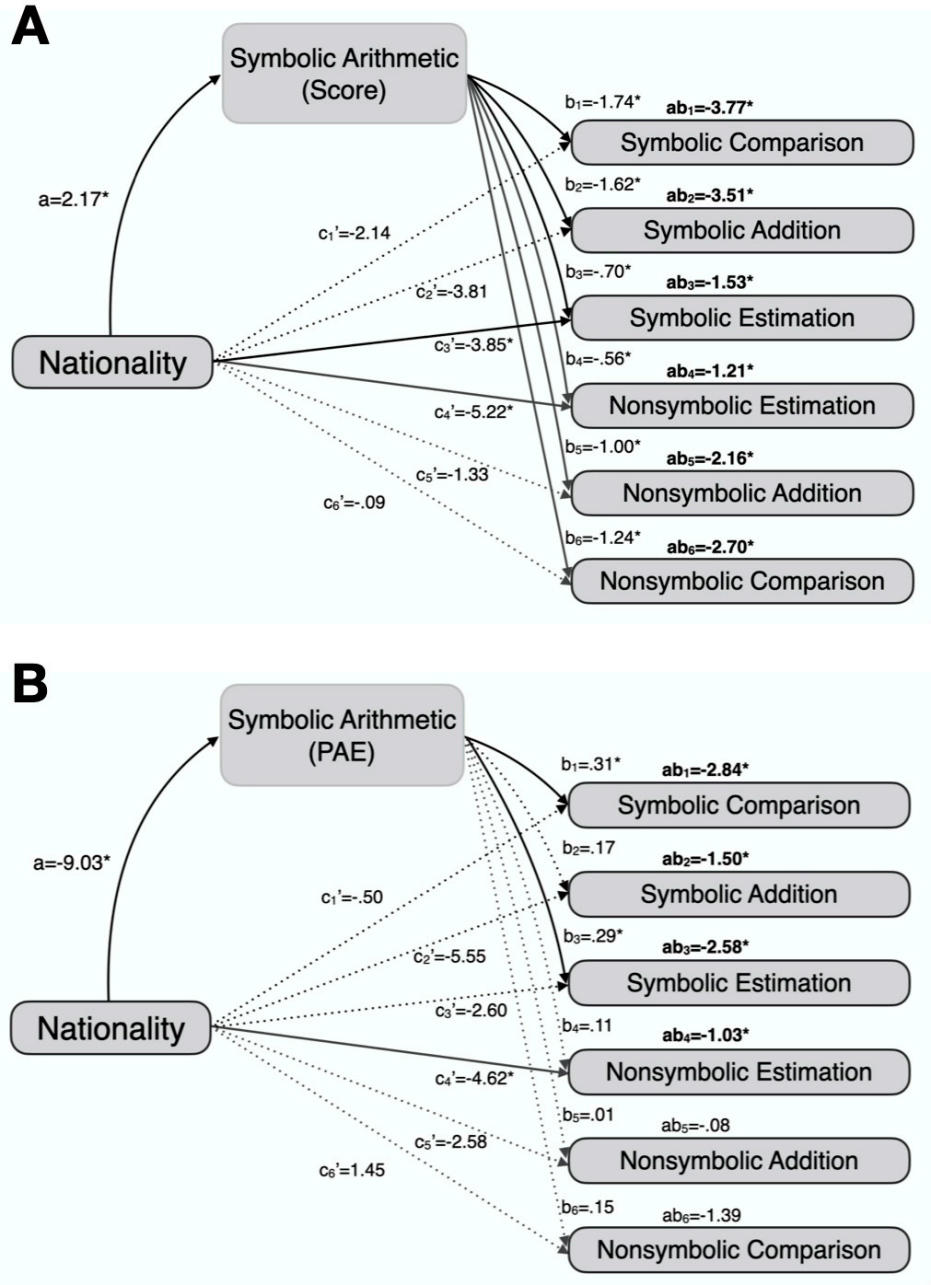
Alternative mediations. Solid and dotted lines represent significant and non-significant relations between variables respectively. Symbolic arithmetic scores are the significant mediator of the effect of nation on all six numerical magnitude tasks (A), whereas symbolic arithmetic PAEs significantly mediate nationality effects on symbolic-numeric and spatial-numeric tasks (B).

One may argue that the effect of nationality on symbolic arithmetic is not mediated by numerical magnitude knowledge; rather, numerical magnitude knowledge is entirely mediated by symbolic arithmetic. This possibility was tested for in new mediation analyses using two symbolic arithmetic measures. For symbolic arithmetic scores, symbolic arithmetic performance did significantly mediate nationality effects on all six numerical magnitude tasks (ab’s ranged from -3.77 to -1.21, all of which significantly differed from zero; Fig 6A). However, there remained significant direct effects of nationality on numerical abilities, as predicted by our original mediation model. Symbolic arithmetic PAEs mediated nationality effects on symbolic-numeric and spatial-numeric tasks (ab’s ranged from -2.84 to -.08, four of which significantly differed from zero, Fig 6B), but a direct effect of nationality still remained significant on non-symbolic number-line estimation.

## Discussion

Since at least 1967, when large-scale international comparisons of math achievement started, US children have trailed their East Asian peers across a wide range of math tests. However, attempts to account for this finding have not identified specific cognitive mediators by which these inputs exercise their effects. In this paper, we used a multiple mediation analysis to identify specific mechanisms by which national differences in math proficiency may arise.

We hypothesized that national differences in mathematics skills, particularly symbolic arithmetic, arise through national differences in numerical magnitude representations. This possibility implies that if the representations of numerical magnitude of US kindergartners were equivalent to those of their Chinese peers, they might be as good at symbolic arithmetic—in spite of learning English number words, receiving the amount of math instruction provided by US schools, being taught by teachers with American training, and typically valuing mathematics less than Chinese peers.

Our findings supported the hypothesis that nation effects were substantially indirect and that they occurred by means of superior numerical magnitude representations. Mediation analyses revealed that a considerable proportion of the whole nationality effect on symbolic arithmetic (81% for arithmetic scores, 39% for arithmetic PAEs) arose from the indirect paths of numerical magnitude representations, specifically those indexed by symbolic number comparison, symbolic approximate addition, and both symbolic and non-symbolic number-line estimation (81% in the 6-mediator analysis and 80% in the 4-symbolic-and-spatial-mediator analysis for arithmetic scores; 45% in the 6-mediator analysis and 33% in the 4-symbolic-and-spatial-mediator analysis for arithmetic PAEs). The relative importance of symbolic over non-symbolic skills seems straightforward—quality of perception of non-symbolic numerosity seems likely to be a universal cognitive capacity, whereas quality of symbolic number requires cultural learning [49, 50]. The highly similar psychophysical profile of non-symbolic number comparison and addition in Chinese and US children support this view.

It was somewhat surprising, then, that non-symbolic number-line estimation differed strongly between Chinese and American children, as well as that the strength of that difference was correlated with symbolic arithmetic. Any attempt to explain this is admittedly speculative, but it may be that accurately estimating the position of a set on a number line requires skills that are common to the symbolic skills tested (e.g., ability to encode equivalent ratio information, as in the analogy ••••:••::••:•).

Beyond these findings, the current study has several limitations that should be addressed in future research. First, like all cross-cultural studies, many unmeasured differences exist and may contribute to the outcome variables of interest. Second, the study was correlational and was conducted with only a single age group. Including an experimental intervention and tracking its effects over time would provide a more robust test of the mediating effects of numerical magnitude judgments. Further, the current study sought to investigate the cognitive mechanisms of cross-national differences in symbolic arithmetic, but children were recruited only from the US and China. Future work is needed to test whether findings in the present research generalize to comparisons of other nations with different languages, cultures, curricula, and economic status. Lastly, our study involved relatively a small number of children for mediation. Although the power analysis using the Sobel test showed that our study was adequately powered, there is little consensus on the method of power analysis for mediation [29, 51]. As a result, a larger sample may prove to be necessary.

The results from our study closely parallel two sets of prior findings. The first concerns the causal relation of number-line estimation to math proficiency; producing improvements in children’s mapping of numerical magnitude onto a number-line also improved how well children learned answers to arithmetic problems from a math lesson [52]. These results provide experimental evidence that buttresses our mediational analysis. The second set of relevant findings concerns the relation between SES and math proficiency. Like US children in the current study, children from low SES backgrounds within the US population exhibit deficits in representations of numerical magnitudes and math proficiency relative to children from more affluent backgrounds. In addition, interventions that focus on numerical magnitude representations in low SES children have eliminated the differences in math proficiency between low and high SES children (e.g., [15]). Combined with our current results, these results imply that the effect of SES on early math proficiency might also be mediated by the quality of numerical magnitude representations.

Finally, the combination of all three results has an important implication for closing the “learning gap.” Much, perhaps most, of the “learning gap” between US and Chinese students could be closed if educational interventions improved numerical magnitude representations of American children. Experimental studies that examine children’s numerical magnitude knowledge are strongly encouraged.

## Supporting information

S1 Dataset Math assessment and digit coding. (CSV)

S2 Dataset Nonsymbolic and symbolic comparison. (CSV)

S3 Dataset Nonsymbolic and symbolic addition. (CSV)

S4 Dataset Nonsymbolic and symbolic numberline estimation. (CSV)
